# Menetrier's disease in a morbid obese patient undergoing bariatric surgery: A case report

**DOI:** 10.1016/j.ijscr.2022.107539

**Published:** 2022-08-23

**Authors:** Ozan Şen, Şeref Oray, İsmail Çalıkoğlu, Cumhur Özcan, Ahmet Gökhan Türkçapar

**Affiliations:** a Nişantaşı University, Department of Health Sciences, İstanbul, Turkey; b Basaksehir Cam and Sakura City Hospital, Istanbul, Turkey; c Mersin University, Medical Faculty, General Surgery Department, Mersin, Turkey; d Türkçapar Bariatrics, Obesity Center, İstanbul, Turkey

**Keywords:** Bariatric surgery, Preoperative endoscopy, Morbid obesity, Menetrier's disease, Sleeve gastrectomy

## Abstract

**Introduction and importance:**

Menetrier's disease is a rare type of hypertrophic gastropathy characterized by the atrophy of the gastric parietal cells and dilatation of mucus releasing glands. Hereby, we present a morbid obese patient who has undergone laparoscopic sleeve gastrectomy (LSG) and he has also diagnosed with Menetrier's disease.

**Case presentation:**

A 67-year-old male patient whose body mass index (BMI) was 39 kg/m^2^. Preoperative endoscopy was done. There were no pathologies except increased gastric mucosal folds. LSG was done. During the surgery it was noticed that gastric tissue was abnormally thick. After LSG completed, it was observed that there was an abnormal bleeding from the staple line. The staple line was oversewed with 3.0 V-Loc™ and bleeding was stopped. Pathology report was compatible with menetrier's disease.

**Clinical discussion:**

Hypoalbuminemia and *H. pylori* take an important place in diagnosis of Menetrier's disease, but *H. pylori* was not detected and albumin level was normal in our patient. For certain diagnosis full-thickness gastric biopsy is needed. The routine use of preoperative endoscopy in patients scheduled for bariatric surgery was still controversial until recently.

**Conclusion:**

This is the first case with menetrier's disease that has undergone LSG. Preoperative endoscopic evaluation before bariatric surgery is crucial. As in this case, it will be effective in terms performing additional intraoperative precautions when necessary and preventing possible complications.

## Highlights

• Menetrier's disease is a rare type of gastropathy which is characterized by large gastric folds.
• This is the first case who has undergone bariatric surgery and has been diagnosed with menetrier's disease
• Pre-operative endoscopy is crusial in evaluating possible gastric pathologies before bariatric surgery

## 1 Introduction

Menetrier's disease is a rare type of hypertrophic gastropathy characterized by the atrophy of the gastric parietal cells and dilatation of mucus releasing glands. It was first described by the French pathologist Pierre Menetrier. This disease causes increased gastric mucus secretion, hypoclorhydria and in some cases loss of protein [1]. The etiology of the disease is not fully understood. According to one study, *Helicobacter pylori* infection could be related to the cause of this disease [2]. Symptoms of Menetrier's disease are; epigastric pain, bloating, vomiting and peripheral edema in some cases due to low albumin level. Menetrier's disease have a premalignant potential [3]. Medical treatment options of Menetrier's disease are; diet with high protein content, *H. pylori* eradication, cetuximab and octreotide [4], [5]. In cases with severe protein loss, partial or total gastrectomy might be needed [6]. Hereby, we will present a morbid obese patient who has undergone laparoscopic sleeve gastrectomy who also got diagnosed with Menetrier's disease. This case report has been reported in line with SCARE 2020 criteria [17].

## 2 Case presentation and treatment

**Fig. 1:**
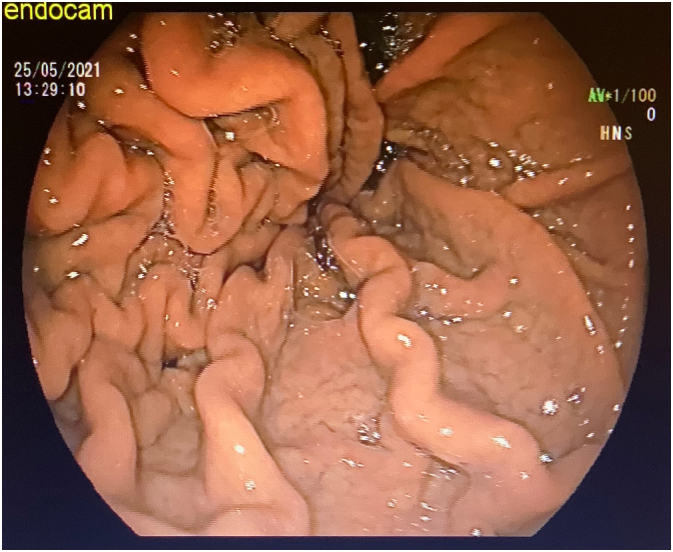
Preoperative endoscopic view.

**Fig. 2:**
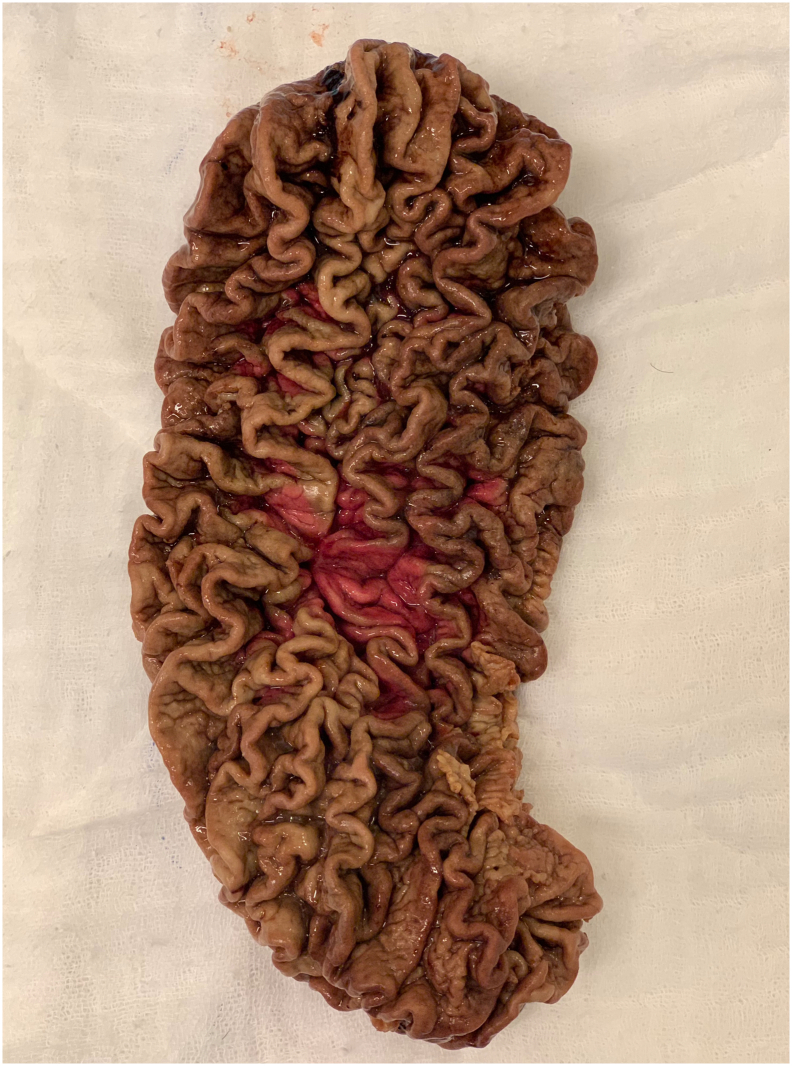
Resected stomach.

**Fig. 3:**
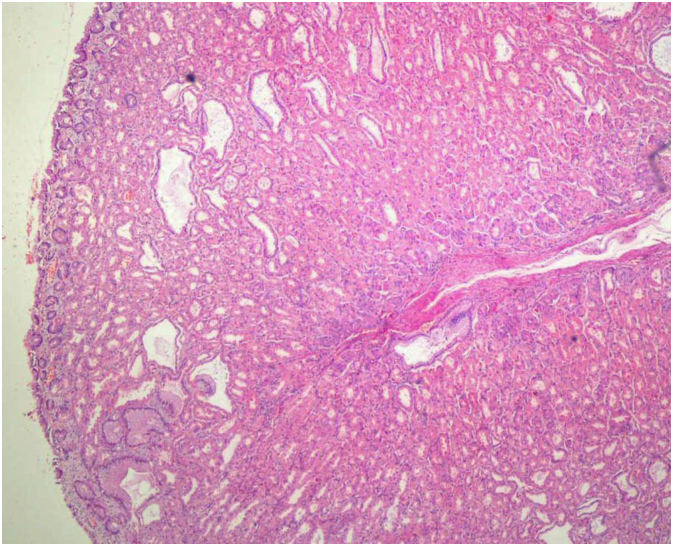
Microscopic view of Menetrier's disease.

Written informed consent was obtained from the patient for this study. A 67-year-old male patient has come to our clinic with morbid obesity. His body mass index was 39 kg/m^2^. Comorbidities of the patient were insulin resistance, dyslipidemia and hepatosteatosis. In preoperative assessment, patient did not state any gastric problems such as gastric ulcer, gastritis or gastroesophageal reflux disease. There was no anemia and albumin level was normal. Laparoscopic sleeve gastrectomy (LSG) was planned for this patient. A preoperative endoscopy as a part of our routine protocol was applied to this patient. In the endoscopy, there were no pathologies except increased mucosal folds in the area of gastric corpus and fundus (Fig. 1). The patient had undergone a surgery following endoscopy. Our surgical technique has been previously described [7]. LSG was performed with 5 trocar technique. During the surgery at gastric stapling period it was noticed that gastric wall was abnormally thick. After sleeve gastrectomy was completed, on the stapler line, it was observed that 3 rows of stapler couldn't close the gastric tissue completely in some regions. Besides, along the entire stapler line there was an abnormal bleeding. As a part of our routine after LSG, the entire stapler line was oversewed with 3.0 V-Loc™. After the hemostasis was completed on the stapler line, resected stomach has been taken out of the abdomen. The resected stomach was opened and analyzed (Fig. 2). In the resected stomach, thickness of gastric wall was 1 cm. The patient had an uneventful postoperative period. Pathology report was compatible with menetrier's disease. Dysplasia or malignancy were not seen (Fig. 3). The patient did not have any problems during the follow-up. Postoperative control endoscopy has not been performed yet after surgery.

## 3 Discussion

Menetrier's disease is a rare disease which is characterized by large gastric folds. Histopathologically, foveolar hyperplasia with glandular atrophia is typical (1). For diagnosis, full-thickness gastric biopsy is needed. Menetrier's disease might be confused with other hypertrophic gastropathic disorders and gastric malignancies. Even though hypoalbuminemia takes an important place in diagnosis, [1] albumin level was normal in our patient. Another agent that may be related to this disease is *Helicobacter Pylori* [2]. In our patient, *H. pylori* was not detected.

Endoscopy before bariatric surgery is important in terms of revealing gastric pathologies that may change the treatment options. In patients scheduled for bariatric surgery, the rate of endoscopic findings leading to a change in surgical treatment is around 8 % [8]. In one of our studies, we found this rate to be 13 % [9]. In addition, we found abnormal findings on endoscopy in 65 % of asymptomatic individuals [9]. The incidence of gastric premalignant and malignant lesions in obese patients is 0.4–0.8 %, which is quite high compared to the general population [8], [10], [11]. We also detected an early-stage carcinoid tumor on preoperative endoscopy in a patient for whom we planned bariatric surgery [12]. The routine use of preoperative endoscopy in patients scheduled for bariatric surgery was still controversial until recently. However, in the consensus report recently published by the International Federation of Bariatric Surgery (IFSO) and the American Bariatric Surgery Association (ASMBS), it was emphasized that preoperative endoscopic evaluation should be performed in order to detect possible gastric pathologies in patients undergoing bariatric surgery [13], [14].

In the case of protein-losing gastropathy or dysplasia that is resistant to medical therapy in Menetrier's disease, the best treatment option is partial or total gastrectomy. In our patient, we think that sleeve gastrectomy is appropriate surgical method in terms of treatment of morbid obesity and menetrier's disease. This is the first case that has undergone bariatric surgery and has been diagnosed with menetrier's disease at the same time. We have not found any other similar case in the literature.

LSG has become one of the most frequently used bariatric surgical methods in recent years [15]. The most important complications after LSG are bleeding (<5 %) and leakage (1–3.9 %) [16]. As in this case, the evaluation of possible gastric pathologies with preoperative endoscopy before bariatric surgery will be effective in terms of performing additional intraoperative precautions when necessary and preventing possible complications.

## 4 Conclusion

Pre-operative endoscopy is extremely important in evaluating possible gastric pathologies during bariatric surgery. In cases where the gastric wall tissue is abnormally thick, as in this case, besides choosing the right stapler cartridge, supporting the stapler line can prevent possible complications (bleeding, leakage).

## Provenance and peer review

Not commissioned, externally peer-reviewed.

## Funding

No funding.

## Ethical approval

The patient has given her informed consent for this publication. It is exemption from ethical approval because it is an observation report.

## Consent

Written informed consent was obtained from the patient for publication of this case report and accompanying images. A copy of the written consent is available for review by the Editor-in-Chief of this journal on request.

## Author contribution

Each author have participated sufficiently in the work to take public responsibility for appropriate portions of the content. All authors met all of the following criteria:

- Substantial contributions to the conception or design of the work; or the acquisition, analysis, or interpretation of data for the work; OS, SO, IC, CO and AGT
- Drafting the work or revising it critically for important intel- lectual content; OS
- Final approval of the version to be published; OS, AGT
- Agreement to be accountable for all aspects of the work in ensuring that questions related to the accuracy or integrity of any part of the work are appropriately investigated and resolved.

AT and OS operated the patient.

OS wrote the first draft of the manuscript.

OS and AT wrote the final draft of the manuscript.

OS, SO, IC and CO made the corrections in English. All authors have read and approved the final report

## Registration of research studies

Not applicable.

## Guarantor

Ozan Sen.

## Declaration of competing interest

No conflict of interest.
